# Synergism of ursolic acid and cisplatin promotes apoptosis and enhances growth inhibition of cervical cancer cells via suppressing NF-κB p65

**DOI:** 10.18632/oncotarget.22133

**Published:** 2017-10-30

**Authors:** Lan Li, Yu Hou, Jing Yu, Yulin Lu, Li Chang, Meiping Jiang, Xingrao Wu

**Affiliations:** ^1^ Department of Radiation Oncology, The Third Affiliated Hospital of Kunming Medical University, Cancer Hospital of Yunnan Province, Kunming 650118, China; ^2^ Department of Gynaecology, The Third Affiliated Hospital of Kunming Medical University, Cancer Hospital of Yunnan Province, Kunming 650118, China; ^3^ Nursing School, Kunming Medical University, Kunming 650118, China

**Keywords:** cervical cancer, ursolic acid, cisplatin, NF-κB p65, proliferation

## Abstract

**Objective:**

This study was designed to investigate the effect of combination of ursolic acid (UA) with cisplatin (DDP) on cervical cancer cell proliferation and apoptosis.

**Methods:**

The mRNA and protein expressions of nuclear factor-kappa B (NF-κB) p65 in cervical cancer cells were examined using RT-PCR and western blot. MTT and colony formation assays were performed to examine the DDP toxicity and the proliferation ability of cervical cancer cells. Cell morphology was observed by means of Hoechst33258 and transmission electron microscopy (TEM). The apoptosis rate and cell cycle were assessed through flow cytometry assay. Western blot was used to detect the expression of apoptosis-related molecules.

**Results:**

The mRNA and protein expressions of NF-κB p65 in cervical cancer cells were significantly higher than that in cervical epithelial cells. The combined treatment of UA and DDP inhibited cervical cancer cell growth and promoted apoptosis more effectively than DDP treatment or UA treatment alone (*P* < 0.05). Compared with the DDP group and UA group, the expressions of Bcl-2 and NF-κB p65 in DDP +UA group were decreased, while the expressions of Bax, Caspase-3 and PARP cleavage were observably increased. The expression of nuclear NF-κB p65 significantly reduced in UA group and DDP +UA group. si-p65 group displayed a decrease of cell proliferation ability and led to a significant reduction in the number of SiHa cell colony formation.

**Conclusion:**

The combination of UA with DDP could more effectively inhibit SiHa cells proliferation and facilitate cell apoptosis through suppressing NF-κB p65.

## INTRODUCTION

Cervical cancer is considered one of the most common female diseases and also a major cause of cancer-related death in female populations [1]. Advanced stage cervical cancer cells are extremely invasive, which is more likely to induce cancer cell metastasis and increases the mortality rate of the cancer [2]. Current therapeutic approaches of the cervical cancer include surgery, chemotherapy and radiotherapy which may be used individually or in combination with other approaches [3, 4]. However, both surgical removal and radiotherapy can incur long-term complications or sequela, which may exert negative influence on the prognosis of cervical cancer patients [5].

Ursolic acid (UA), a natural pentacyclic triterpene compound, is extracted from various herbal plants including *Ligustrum lucidum* and *Eriobotrya japonica* [6]. Like other triterpenoids, UA possesses anti-oxidation, anti-microbial, anti-inflammation and anti-tumor properties [7, 8]. Current research has indicated that UA might have an inhibitive function on tumorigenesis and tumor growth [9, 10]. Furthermore, UA has been found to induce apoptosis in cervical carcinoma cells [11], prevent the proliferation of colorectal cancer cells [12] and induce breast cancer cell apoptosis [13]. Although the anti-cancer function of UA has been widely studied, the explicit anti-cancer mechanism of UA remains unknown.

Cisplatin (DDP) is a cell cycle non-specific antineoplastic drug, which is applicable for the treatment of several types of cancers and it is also recommended to applied to chemotherapy for epithelial malignancies, such as lung cancer [14], ovarian cancer [15], testicular cancer [16] and cervical cancer [17]. DDP and its derivatives have been found to have encouraging anti-cancer effects on different types of cancers [18]. DDP-based chemotherapy along with radiotherapy is the most widely accepted approach for the treatment of cervical cancer [19], but the effectiveness of conventional chemotherapy is still limited [20]. Therefore, many researchers encourage the combined method of chemotherapies with multiple therapeutic drugs to improve overall treatment efficacy. Additionally, DDP is an efficacious anti-tumor agent and exerts cytotoxic effects on cancer cells and promotes cancerous cell apoptosis. Moreover, DDP is found to have the capability to induce the activation of Nuclear factor-kappa B (NF-κB) in cancer cells [21].

NF-κB is a family of transcription factors which play a significant role in the regulation of diverse genes involved in cell proliferation, inflammation, immune response and oncogenesis [22]. The activation of NF-κB, which is induced by chemotherapeutic compounds in cancer cells, has a negative impact on the treatment efficiency of cancer [23]. It has been reported that NF-κB is constitutively activated in high-grade squamous intraepithelial lesions and squamous cell carcinomas of human uterine cervix [24]. Numerous previous studies suggested that NF-κB activation not only contributes to the migration and invasion of cancer cells, but also affects cell survival and gene expressions related to tumor proliferation and metastasis [25-27]. Five subunits of NF-κB have been identified, namely, gp105/p50 (NF-B1), p100/p52 (NF-B2), p65 (RelA), RelB, and c-Rel [28]. The most common and best-characterized form of NF-κB is the p50/p65 heterodimer, which is widely expressed in the CNS and plays an important role in the regulation of gene expression [29]. In the current study, we studied on the effect of UA on NF-κB p65. We hypothesized that UA may be able to inhibit NF-κB p65 activation [30]. Until now, little evidence of the synergism between UA and DDP in the treatment of human cervical cancer has been revealed. Therefore, we carried out this study in order to clarify the synergistic anti-cancer effect of UA and DDP on human cervical cancer cells. We suspected that UA coupled with DDP may offer superior therapeutic effects on human cervical cancer.

## RESULTS

### NF-κB p65 expression was up-regulated in cervical cancer cells

**Figure 1:**
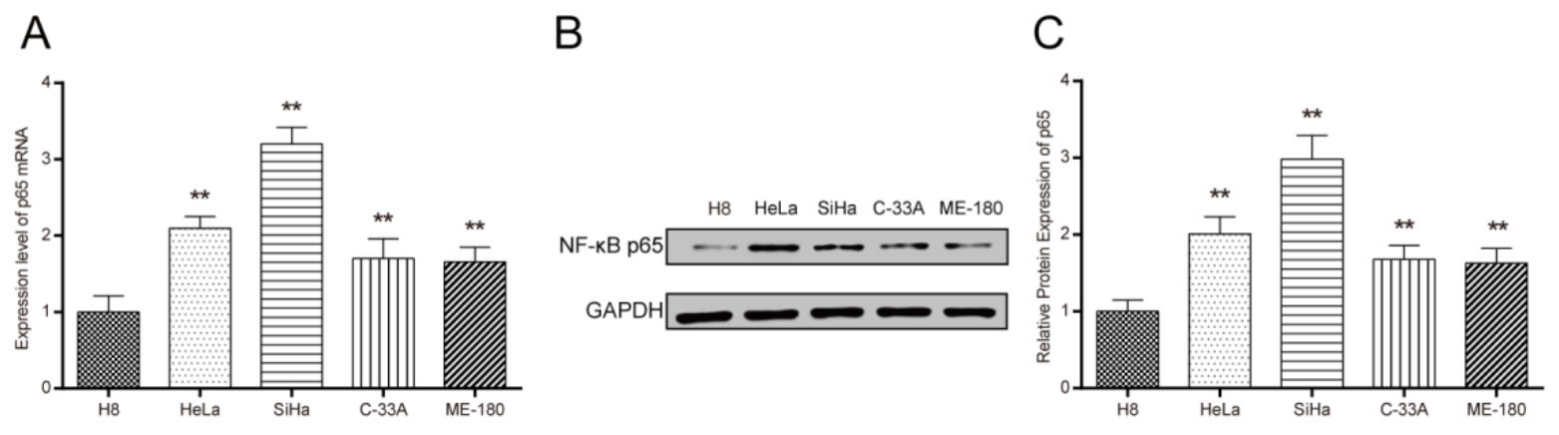
The expression of NF-κB p65 in different cervical cancer cell lines and human cervical epithelial cells H8 **(A)** The mRNA expression of NF-κB p65 in different cervical cancer cell lines detected by RT-PCR. **(B-C)** Western blot detected the protein expression of NF-κB p65 in different cervical cancer cell lines. The expression of NF-κB p65 in cervical cancer cells was higher than that in the cervical epithelial cells H8. ** *P* < 0.01 versus control group. Control: cervical epithelial cells primary cultured from uterine tissues.

Cells were collected at logarithmic growth period. NF-κB p65 expression was detected using RT-PCR and western blot. The mRNA expression level of NF-κB p65 was significantly increased in cervical cancer cell lines HeLa, SiHa, C-33A and ME-180 when compared with human cervical epithelial cells H8(Figure 1A, all *P* < 0.01). As shown in Figure 1B and 1C, the protein expression level of NF-κB p65 was consistent with the trend of the NF-κB p65 mRNA expression. Notably, SiHa cells presented a relatively high expression in NF-κB p65.

### UA enhanced DDP-induced proliferation inhibition of cervical cancer cells

**Figure 2:**
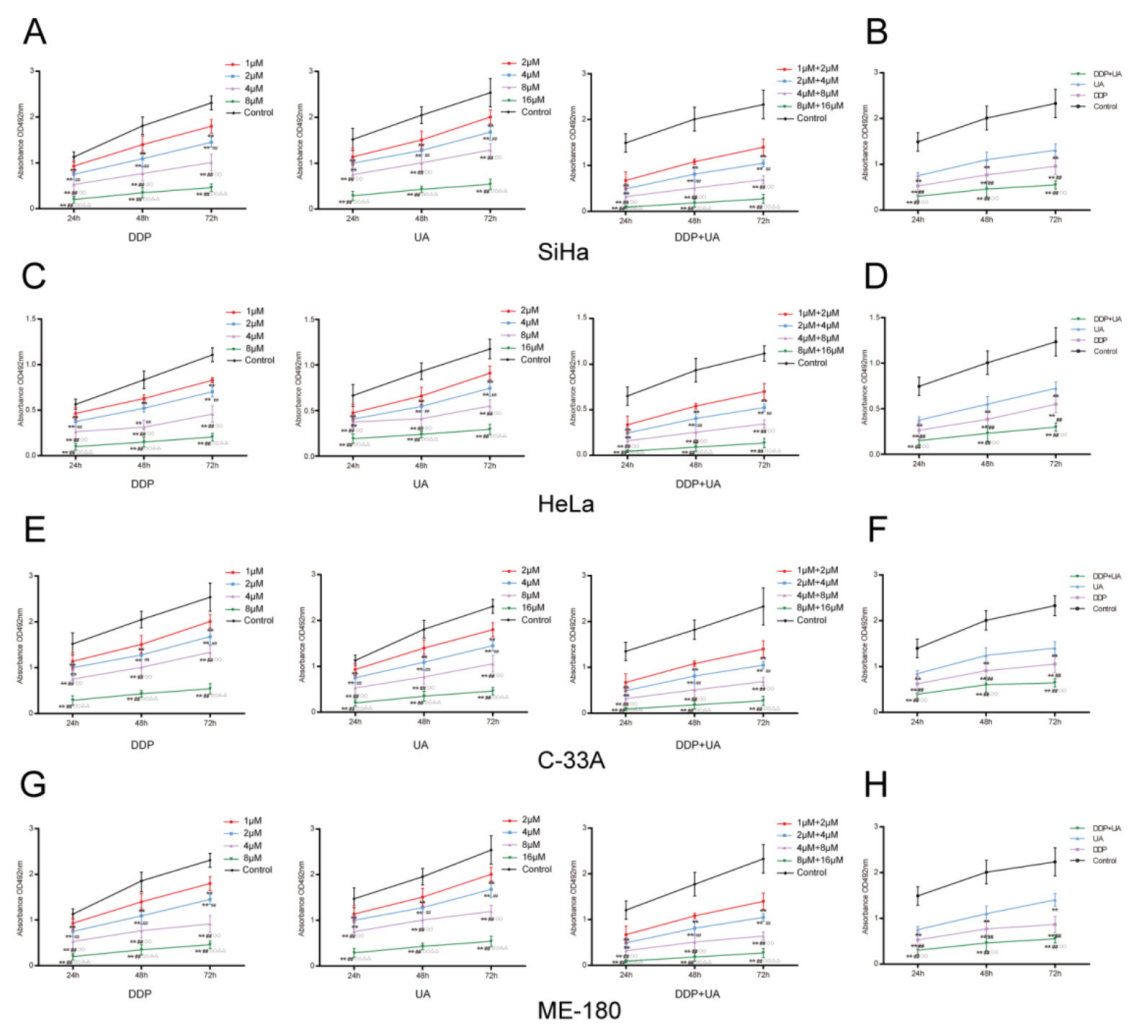
UA enhanced DDP-induced proliferation inhibition of cervical cancer cell lines Cell proliferation was measured by MTT assay in SiHa **(A)**, HeLa **(C)**, C-33A **(E)** and ME-180 **(G)** cell lines. Results (mean ± SD) were from six independent experiments. The joint treatment of DDP and UA reduced the proliferation of cervical cancer cell lines compared with the DDP or UA treatment solely. (A, C, E, G) ** *P* < 0.01 versus control group; ^##^ *P* < 0.01 compared with the 1 μM DDP group; ^○○^ *P* < 0.01 compared with the 2 μM DDP group; ^ΔΔ^ *P* < 0.01 compared with the 4 μM DDP group. ** *P* < 0.01 versus control group; ^##^ *P* < 0.01 compared with the 2 μM UA group; ^○○^ *P* < 0.01 compared with the 4 μM UA group; ^ΔΔ^ *P* < 0.01 compared with the 8 μM UA group. ** *P* < 0.01 versus control group; ^##^ *P* < 0.01 compared with the 1 μM DDP + 2 μM UA group; ^○○^ *P* < 0.01 compared with the 2 μM DDP + 4 μM UA group; ^ΔΔ^ *P* < 0.01 compared with the 4 μM DDP + 8 μM UA group. **(B, D, F, H)** ** *P* < 0.01 versus control group; ^##^ *P* < 0.01 compared with the DDP group; ^○○^ *P* < 0.01 compared with the UA group. DDP: cisplatin; UA: ursolic acid.

**Figure 3:**
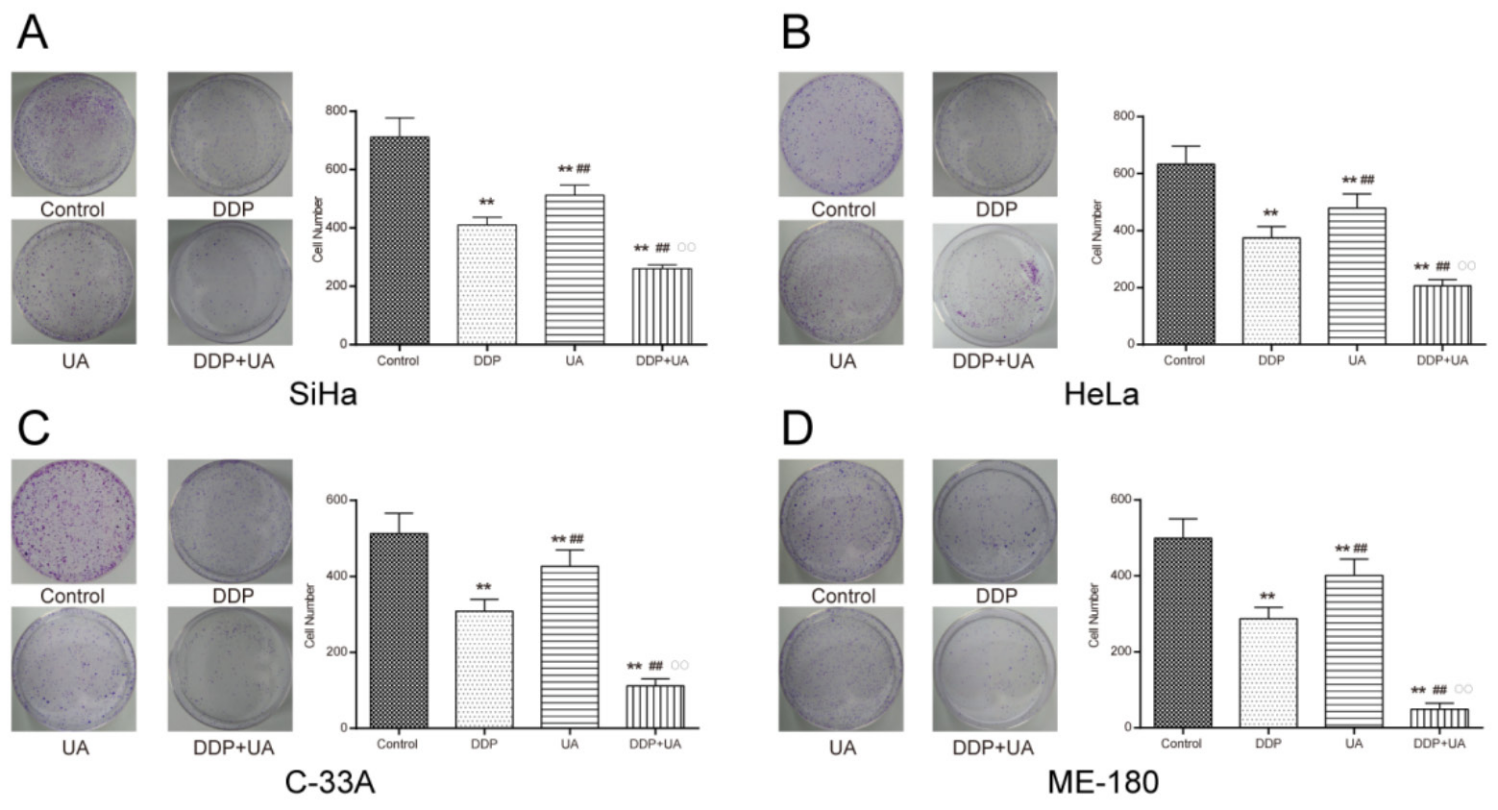
UA enhanced DDP-induced proliferation inhibition of cervical cancer cell lines **(A)** DDP and UA affected cell colony formation of SiHa, **(B)** HeLa, **(C)** C-33A and **(D)** ME-180 ovarian cancer cells. Results (mean ± SD) were from six independent experiments. ** *P* < 0.01 versus control group; ^##^ *P* < 0.01 compared with the DDP group; ^○○^ *P* < 0.01 compared with the UA group. DDP: cisplatin; UA: ursolic acid.

The proliferation of cervical cancer cells was observed by MTT assay for activity and colony formation assay for amount. As suggested by MTT assay, a dose-dependent effect was clearly observed in DDP-treated SiHa cells and the proliferation rate decreased as the dosage increased. Similarly, there was a dose-response effect in cells treated with UA. DDP in conjunction with UA also exhibited a similar dose-response impact on cells (Figure 2A). To avoid the increase of necrotic cells caused by excessive concentration, 4μM DDP, 8μM UA and 4μM DDP + 8μM UA were selected for subsequent experiments. The joint treatment of DDP and UA significantly reduced the proliferation of SiHa cells compared with the DDP or UA treatment solely (all *P* < 0.01, Figure 2B). Colony formation assay was also conducted to test the proliferation of SiHa cells treated with DDP or UA. As shown in Figure 3A, dosing groups displayed a prominent reduction in the colony formation of SiHa cells in comparison with the control group. Compared with the DDP or UA single treatment groups, the combined treatment of DDP and UA significantly diminished the colony formation capabilities of SiHa cells (all *P* < 0.01). Other cell lines showed the same trend in SiHa cells, though the proliferation of cells in other cell lines was lower than in SiHa cells (Figures 2C-2H and 3B-3D, all *P* < 0.01). All of these results revealed that UA could increase DDP-induced proliferation inhibition of cervical cancer cells. The experimental results of all cell lines have no connection with HPV phenotype and the SiHa cells were selected for subsequent experiments objects due to their higher sensitivity.

### UA promoted DDP-induced SiHa cell morphologic changes

**Figure 4:**
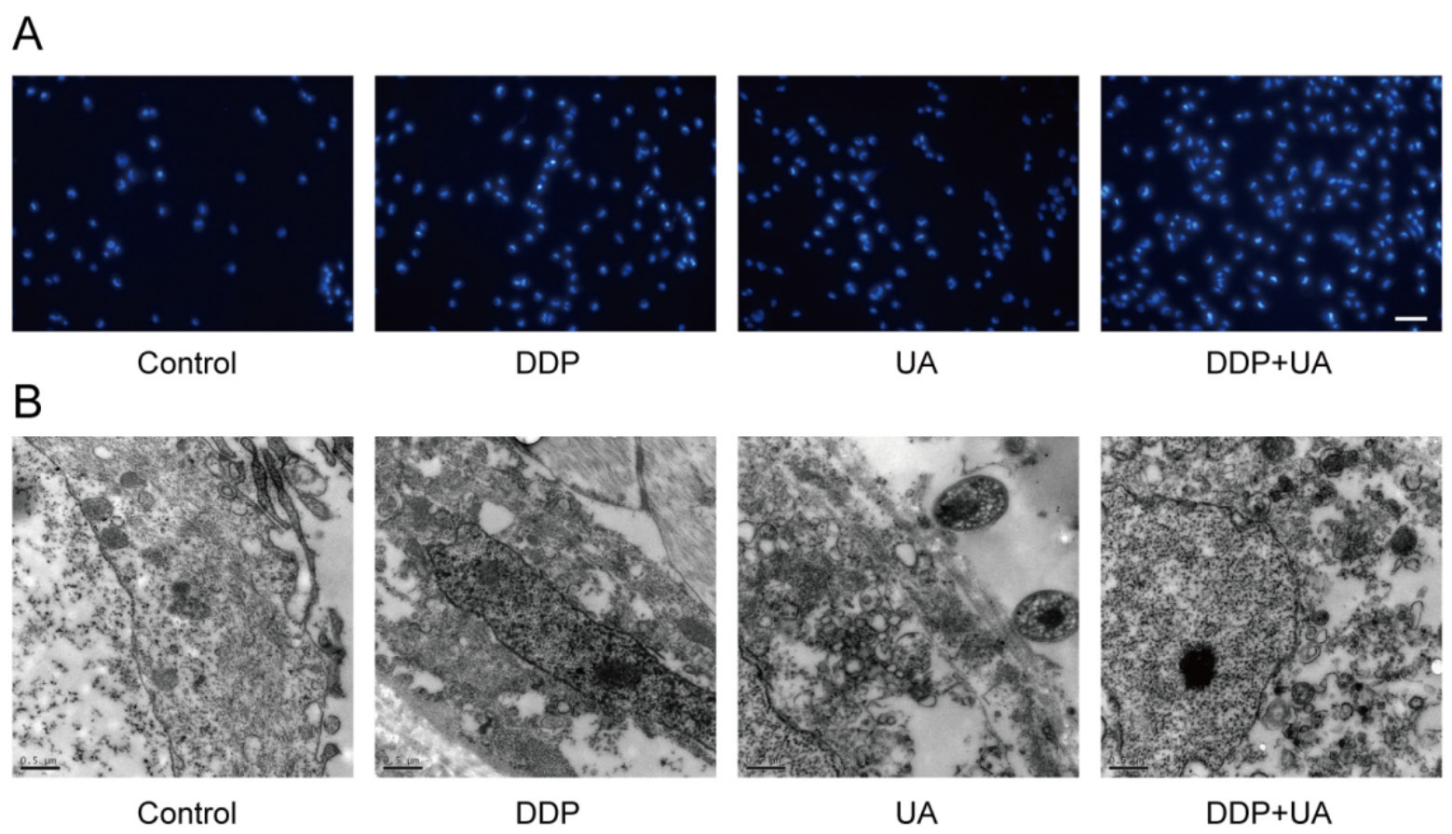
UA promoted DDP-induced SiHa cell morphological changes **(A)** Determination of apoptosis in the SiHa cells 24h post DDP in the presence of UA was analyzed by Hoechst 33258 staining method (×200). **(B)** The ultrastructural morphological investigation of SiHa cells using TEM 72h after DDP treatment in the presence of UA (0.5μm). DDP: cisplatin; UA: ursolic acid.

The nuclei of apoptotic cells and typical apoptotic bodies stained by Hoechst33258 were observed under fluorescence microscopy. Nuclear condensation and fragmentation were observed at 24h after DDP therapy with UA (Figure 4A). Transmission electron microscopy (TEM) showed integrity of cell membrane in the control group. The cells in the dosing groups, particularly in combination DDP + UA treatment group, presented apoptotic features of cells, such as cell shrinkage, nuclear chromatin concentration and broken cell membranes with nuclear lysis (Figure 4B). These results demonstrated that the ultrastructure of SiHa cells was severely damaged under the joint influence of DDP and UA.

### The combination UA with DDP significantly promoted apoptosis of SiHa cells

**Figure 5:**
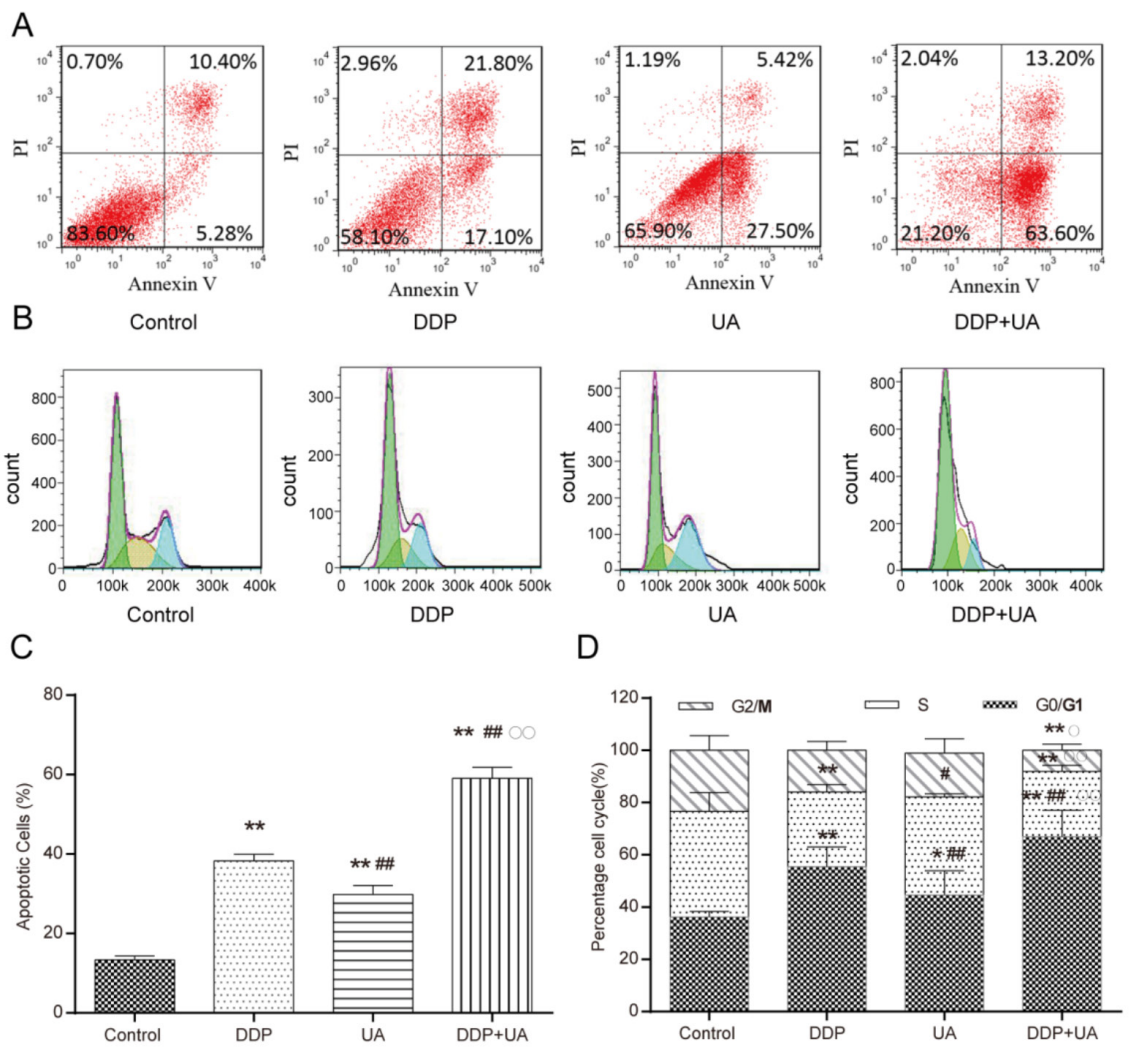
UA stimulated DDP-induced cervical cancer cell apoptosis **(A-B)** The apoptosis status of cells after 24h treatment was detected via flow cytometric analysis. The apoptosis percentage of SiHa cells treated with DDP and UA reach to 38.9% and 32.92% respectively. The apoptosis percentage of combined treatment of DDP and UA reach to 76.8%. **(C-D)** DDP and UA affected cell-cycle of SiHa cells. Results (mean ± SD) were from six independent experiments. The number of cells arrested in G0/G1 in control, DDP, UA and DDP+UA are 38%, 57%, 50% and 70% respectively. The number of cells arrested in S phase in control, DDP, UA and DDP+UA are 40%, 25%, 30% and 20% respectively. The number of cells arrested in G2/M in control, DDP, UA and DDP+UA are 22%, 18%, 20% and 10% respectively. * *P* < 0.05, ** *P* < 0.01 versus control group; ^#^ *P* < 0.05, ^##^ *P* < 0.01 compared with the DDP group; ^○^ *P* < 0.05, ^○○^ *P* < 0.01 compared with the UA group. DDP: cisplatin; UA: ursolic acid.

Apoptotic cells were detected by flow cytometry. Results from flow cytometry analysis showed that the apoptosis percentage (mean ± SD) of SiHa cells treated with DDP (12.29 ± 0.66) % was significantly increased when compared with those treated with UA (9.87 ± 0.31) % (*P* < 0.01), whereas that of cells in the control group was significantly lower (4.11 ± 0.98) % (both *P* < 0.01). For SiHa cells, the combined treatment of DDP and UA significantly elevated the apoptotic rate of the cells in comparison with either DDP treatment group or UA treatment group (*P* < 0.01) (Figure 5A and 5B). As shown in Figure 5C and 5D, cell mitosis was suppressed and most of cells were arrested in G0/G1 in dosing groups, especially in DDP+UA group (all *P* < 0.05), the percentage of apoptotic cells exhibit in Supplementary Table 1. These findings demonstrated that the combined treatment of UA with DDP further induced the apoptosis of cervical cancer cells.

### The expression of apoptosis-related molecules and cell cycle factors

**Figure 6:**
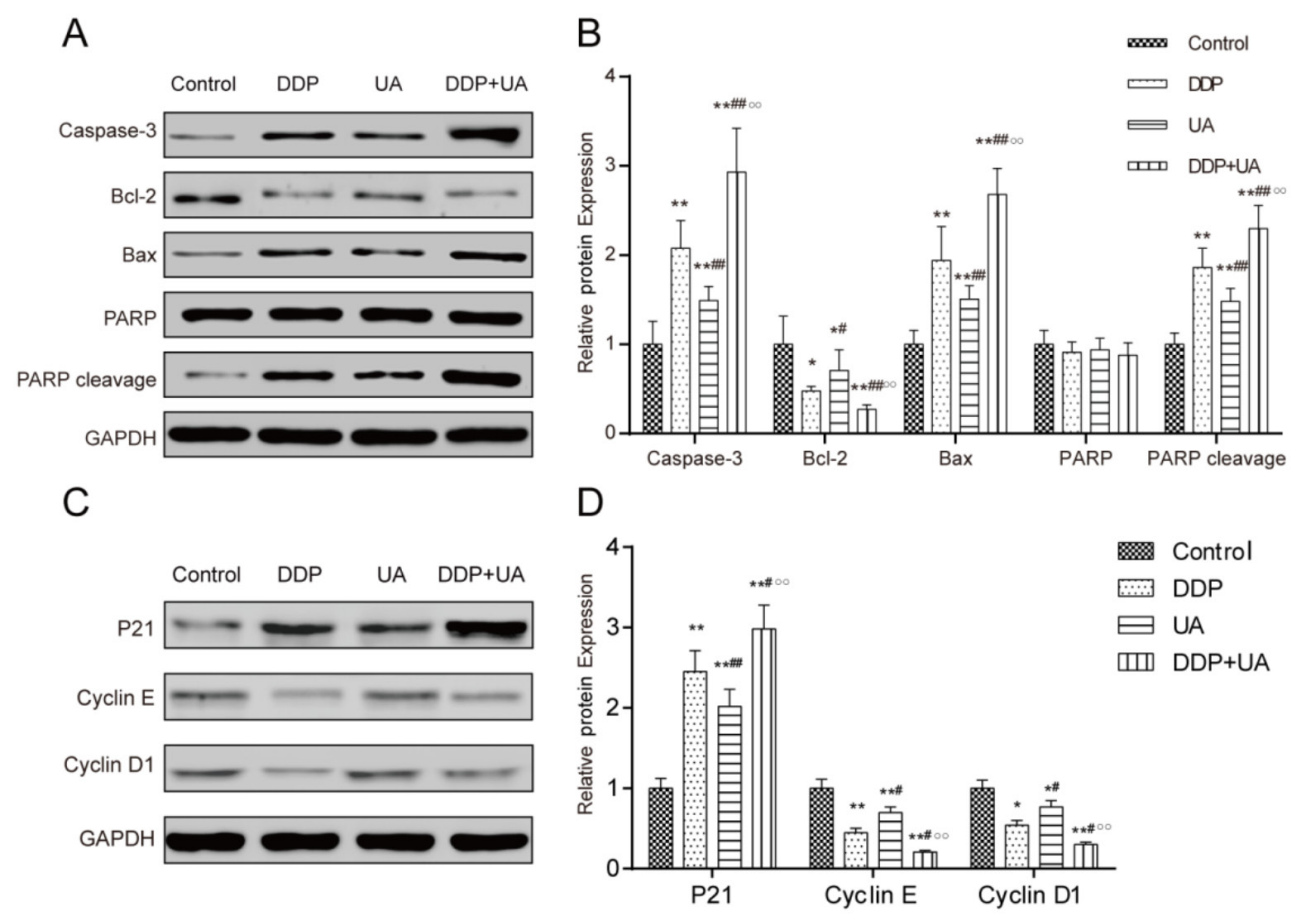
RT-PCR and western blot were used to test the expression levels of apoptosis-related molecules and cell cycle factors **(A)** The expressions of apoptosis-related molecule (Caspase-3, Bcl-2, Bax, PARP and PARP cleavage) in SiHa cells were evaluated by western blot analysis. **(B)** The expressions of apoptosis-related molecule (Caspase-3, Bcl-2, Bax, PARP and PARP cleavage) in SiHa cells were evaluated byRT-PCR. **(C)** The expressions of cell cycle factors (p21, cyclin D1 and cyclin E) in SiHa cells were evaluated by western blot. **(D)** The expressions of cell cycle-related molecule factors (p21, cyclin D1 and cyclin E) expressions in SiHa cells were evaluated by RT-PCR. Results (mean ± SD) were from six independent experiments. * *P* < 0.05, ** *P* < 0.01 versus the control group;^#^ *P* < 0.05, ^##^ *P* < 0.01 compared with the DDP group; ^○○^ *P* < 0.01 compared with the UA group. DDP: cisplatin; UA: ursolic acid.

Western blot was used to detect the expression of apoptosis-related molecules, including Bcl-2, Bax, caspase-3 and PARP. The expression level of Bcl-2 was significantly down-regulated while the expressions of Bax and caspase-3 as well as PARP cleavage were significantly up-regulated in DDP or UA treatment groups when compared with the control group (all *P* < 0.05). Furthermore, the expression level of Bcl-2 in the cells treated with both DDP and UA significantly decreased, whereas the expressions of Bax, caspase-3 and PARP cleavage significantly increased in comparison with the DDP treatment group (all *P* < 0.05) (Figure 6A and 6B, Supplementary Table 2).

We speculated that the cell cycle changes were incurred by the changing cell cycle factors. To verify our hypothesis, western blot was used to examine the protein expression levels of the cell cycle factors. The results displayed that the DDP group and UA group had considerably higher p21 expression and lower expressions of cyclin D1 and cyclin E than control group (all *P* < 0.05). The expression of p21 in DDP + UA group dramatically increased, whereas the expressions of cyclin D1 and cyclin E significantly decreased compared with those in DDP group (all *P* < 0.05) (Figure 6C and 6D, Supplementary Table 3).

### UA restrained DDP-induced NF-κB p65 activation in SiHa cells

**Figure 7:**
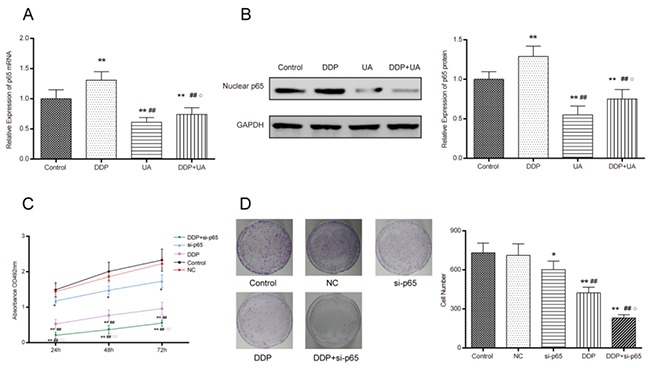
UA restrained DDP-induced NF-κB p65 activation in SiHa cells **(A)** The expression level of the mRNA nuclear NF-κB p65 was measured by RT-PCR. **(B)** The expression level of the protein nuclear NF-κB p65 was measured by western blot and RT-PCR. Results (mean ± SD) were from six independent experiments. ** *P* < 0.01 versus the control group; ^##^ *P* < 0.01 compared with the DDP group; ^○^*P* < 0.05 compared with the UA group. **(C)** Cell proliferation was measured by MTT assay. **(D)** DDP and si-p65 affected cell colony formation in SiHa cells. Results (mean ± SD) were from six independent experiments. * *P* < 0.05 versus control group; ^**^P < 0.01 versus the control group; ^##^ *P* < 0.01 compared with the si-p65 group; ^○^*P* < 0.05 compared with the DDP group. NC: cells transfected with empty vector plasmid. si-p65: cells transfected with vector of si-p65 sequences. DDP + si-p65: cells transfected with vector of si-p65 sequences and treated with DDP. DDP: cisplatin; UA: ursolic acid.

We also assessed the influence of UA on DDP-induced NF-κB p65 activation in SiHa cells. As suggested by Figure 7A and 7B and Supplementary Table 4, the expression of nuclear NF-κB p65 significantly reduced in UA group and DDP +UA group in comparison with the DDP group (all *P* < 0.05). MTT and colony formation assays were employed to examine DDP toxicity. Compared with control group, si-p65 group displayed a decrease of cell proliferation ability (*P* < 0.05). Moreover, the proliferation of cells in the DDP + si-p65 decreased more dramatically than that in DDP group (*P* < 0.01). There was no significant difference between control group and NC group (Figure 7C). As shown in Figure 7D, si-p65 led to a significant reduction in the number of SiHa cell colony formation in comparison with the control group (*P* < 0.05). Furthermore, the combined treatment of DDP and si-p65 diminished the colony formation capabilities of SiHa cells more significantly than DDP group (all *P* < 0.01). Similarly, there was no significant difference between control and NC groups. All the above results indicated that UA could inhibit DDP-induced NF-κB activation in SiHa cells.

## DISCUSSION

Cervical cancer is one of the most disturbing health issues in females and it is also the second most common cause of cancer-related death in female population around the world [1]. But both surgical removal and radiotherapy can bring about long-term complications or sequela, which give rise to negative influence on the prognosis of cervical cancer patients [1]. In our study, we tried to figure out the anti-cancer function of UA, DDP and the combination UA and DDP on cervical cancer cells. A large number of studies have reported that NF-κB which play a significant role in the regulation of diverse genes involved in cell proliferation, inflammation, immune response and oncogenesis [22]. The p50/p65, the member of NF-κB, is widely expressed in the CNS and plays an important role in the regulation of gene expression. In the current study, we studied on the effect of UA on NF-κB p65 firstly.

MTT assay, colony formation assay and flow cytometry assay revealed that UA augmented the DDP-induced cell proliferation inhibition and facilitated apoptosis of cervical cancer cells via the suppression of NF-κB p65 activation. Conventional chemotherapies, including platinum-based and non-platinum-based regimens, are currently applied to the treatment of cervical cancer. These chemotherapies are associated with strong non-specific cytotoxicity, restricted therapeutic indices, adverse effects and chemo-resistance to single agents [20, 31]. Therefore, the treatment of chemotherapies combined with several agents is considered a promising therapeutic strategy, which can help enhance the efficacy and minimize the adverse effects of cervical cancer treatment.

Many chemotherapeutants like DDP can exert cytotoxic effects on cancer cells by inducing cell apoptosis. DDP also can activate a significant transcription factor NF-κB in the process of human tumor pathogenesis [32]. We determined the proliferation of human cervical cancer cells to reflect DDP toxicity after NF-κB p65 was silenced. We also found that exogenous expression of NF-κB reduced the toxicity of DDP. Combined with the results of the mRNA and protein expression levels of NF-κB p65, we came to the conclusion that the increased apoptosis induced by UA in DDP treated cervical cancer cells was due to NF-κB suppression. This result verified the findings reported by Xing *et al.* that NF-κB is responsible for tumor cell survival, tumor cell growth and direct activation of anti-apoptotic gene products [33]. Atoxigenic NF-κB suppressors may contribute to a reduced anti-apoptotic threshold of drug resistance, potentially enlarging the sensitivity of medications and thus the suppressive effects of medications on tumor cells may become more significant [34]. A large number of studies have reported that the overall effectiveness of treatments for cervical cancer is likely to be enhanced through the combination of various chemotherapy agents [34, 35]. Previous studies have reported that the combination of UA17 (an UA derivative) and DDP could enhance cell growth inhibition via the induction of cell cycle arrest at S phase and G1/S transition, as well as apoptosis [36]. This finding was consistent with our results as presented in Figure 5. We also verified our hypothesis that the causes of the changes in cell cycles were the altered cell cycle factors including p21, cyclin E and cyclin D1.

Our study showed that the combination of UA and DDP down-regulated Bcl-2 expression levels and up-regulated the expression levels of Bax and caspase-3. This indicated that DDP-induced cervical cancer cell apoptosis was enhanced via the down-regulation of Bcl-2 expression and the up-regulation of Bax and caspase-3 expressions. UA is a pentacyclic triterpenoid which may produce multifarious biologic activities including hepatoprotective, antibacterial, anti-proliferative and immunomodulatory activities [37]. UA induces the release of apoptosis factors from mitochondrion in M4Beu cells and inhibits tumor progression, tumorigenesis and angiogenesis through the suppression of lipoxygenase, COX-2, MMP-9 and NOS expressions [11, 38]. In addition, UA stimulated the intrinsic apoptotic mechanism characterized by Bax over-expression, Bcl-2 under-expression and the breakdown of mitochondrial membrane potential [9]. This intrinsic apoptotic mechanism also includes caspase-9-mediated cascade reaction which culminates in caspase-3 activation, a significant mediator of cell apoptosis [39].

In the current study, only one human cervical cancer cell line SiHa (HPV 16 positive) was used in our study and *in vivo* experiment was lacked in our study, thus the influence of UA and DDP on cervical cancer cell proliferation and apoptosis may not be completely representative. Additionally, whether the effects of UA and DDP have synergistic effects or additive effects has not been fully explored. Therefore, there still exist some limitations in this study, which need to be addressed in future researches. The molecular mechanism of UA in combination with DDP in the treatment of cervical cancer needs to be further investigated.

In summary, the synergism of UA and DDP could significantly induce cell apoptosis and enhance growth inhibition of human cervical cancer cells by suppressing NF-κB p65 activation. The findings suggested that the combined approach of UA with DDP may generate better therapeutic effects on cervical cancer compared with conventional treatments. UA might be a novel adjuvant therapeutic drug for the treatment of cervical cancer.

## MATERIALS AND METHODS

### Materials and cell culture

Ursolic acid (UA, Sigma, USA) and cisplatin (DDP, Sigma) were dissolved in dimethyl sulfoxide (DMSO) and were stored at -20°C. Human cervical cancer cell lines HeLa (HPV 18 positive), SiHa (HPV 16 positive), C-33A (HPV negative) and ME-180 (HPV 68 positive) were obtained from Shanghai Cell Biology Institute of the Chinese Academy of Sciences (Shanghai, China). All cell lines were confirmed using short tandem repeat (STR) profiling (Molecular Diagnostics Laboratory, Dana Farber Cancer Institute). Human immortalized cervical epithelial cells H8, as control group, were purchased from ATCC. All these specimens were obtained from the Third Affiliated Hospital of Kunming Medical University, Cancer Hospital of Yunnan Province. All procedures were carried out in accordance with the Declaration of Helsinki and approved by the Ethics Committee of Human Experimentation of the Third Affiliated Hospital of Kunming Medical University, Cancer Hospital of Yunnan Province. Dulbecco’s Modified Eagles Medium (DMEM) (Gibco, USA) containing 10% fetal bovine serum (FBS; Gibco), streptomycin (100 mg/ml; Gibco) and penicillin (100 units/ml; Gibco) was used to culture cervical cancer cell lines. Roswell Park Memorial Institute medium (RPMI) (Gibco, USA) containing 10% fetal bovine serum (FBS; Gibco) was used to culture human cervical epithelial cells. The cultured cells were incubated at 37°C with 5% CO_2_.

### Cell transfection

The siRNA sequences targeted against p65 were designed using siDESIGN- software (Thermo Scientific, USA) and cloned into pSuper Retro Puro vector (Biovector Science, China). Cells were divided into four groups randomly. Cells in the negative control (NC) group were transfected with empty vector plasmid. Cells in the siRNA-p65 (si-p65) group were transfected with vector of si-p65 sequences. Cells treated with DDP as well as si-p65 were regarded as DDP + si-p65 group. The sequence of si-p65 was shown in Table 1.

**Table 1:** Primers sequences used for quantitative RT-PCR

Genes		Primer pair sequences
p65 siRNA	F	5’-ATAGGATCCCCGGAGAAACGTAAAAGGACATTCAAGAGATGTCCTTTTACGTTTCTCCTTTTTAAGCTTATA-3’
	R	5’-TATAAGCTTAAAAAGGAGAAACGTAAAAGGACATCTCTTGAATGTCCTTTTACGTTTCTCCGGGGATCCTAT-3’
p65	F	5’-AGCTCTAGAGCCATGGACGAACTGTTCCC-3’
	R	5’-CTGTGGATGCAGCGGTCC-3’
GAPDH	F	5’-GAGCCAAAAGGGTCATCATC-3’
	R	5’-TAAGCAGTTGGTGGTGCAGG-3’

F: forward primers; R: reverse primers

### RNA extraction and RT-PCR

Relative expression levels of NF-κB p65 mRNA were detected by RT-PCR. Total RNA isolation from cells was conducted using TRIzol reagent kit (Invitrogen, USA) following manufacturer’s instructions. Complementary DNA (cDNA) was acquired using the Omniscript reverse transcription kit (Qiagen, Germany). Real-time quantitative RT-PCR was conducted by means of ABI7500 quantitative PCR system (Applied Biosystems, USA) to detect the relative mRNA expression level of NF-κB p65. Relevant primer sequences (Invitrogen) were shown in Table 1. The relative expression of p65 mRNA was calculated using the 2^-ΔΔC^ method and normalized to the expression of U6 snRNA. All the above assays were repeated three times.

### MTT cell proliferation analysis

Cell proliferation was evaluated using MTT [3-(4, 5-dimethylthiazol-2-yl)-2, 5-diphenyl-tetrazolium bromide] assay. All cell lines (3×10^3^∼6×10^3^ cells/well) were inoculated onto 96-well plates. The cells were treated with DDP (1, 2, 4 and 8 μM), UA (2, 4, 8 and 16 μM) or combination of DDP and UA, respectively. Cells were separately detected after they were treated at 24 h, 48 h and 72 h. MTT (20 μl, 5mg/ml, Sigma) was added into each well and the result was incubated for another 4 hours at 37 °C with 5% CO_2_. The supernatant was removed from the 96-well plates. Subsequently, DMSO (150 μl) was added into each well and cells were shaken slightly for 10 min in order to dissolve the crystals. Samples were inspected by a microplate reader (SpectraMAX Plus, Molecular Devices, Sunnyvale, CA) at 492 nm and tests were conducted at least six times.

### Colony formation assay

Cells in different treatment groups (Control, DDP, UA and DDP+UA) were diluted with Roswell Park Memorial Institute (RPMI) medium and seeded at 500 cells/well in 35mm culture dishes. Cells were cultured for 2-3 weeks in the environment of 37 °C and 5% CO_2_, then the medium was removed and cells were stained with 0.1% crystal violet for 10 min. The number of colonies in each well was counted.

### Hoechst 33258 staining

SiHa cells were seeded into 24-well plates and treated with 4 μM DDP, 8 μM UA, 4 μM DDP + 8 μM UA and 0.1% DMSO as control at 37 °C for 24h. Cells were washed twice with phosphate-buffered saline (PBS) and fixed in methanol/acetic acid (3:1, v/v) for 10 min at 4 °C. The fixative was removed and cells were washed twice with PBS. Cells were then stained with Hoechst 33258 (5 μg/mL, Sigma) for 5 min and then examined under a fluorescent microscope.

### Transmission electron microscopy (TEM)

Cells were grown in 24-well plates, as described above, either with a culture medium supplemented with 4 μM DDP, 8 μM UA and 4 μM DDP + 8 μM UA, or with 0.1% DMSO as control at 37 °C and 5% CO_2_ for 72h. Cells were collected by centrifugation at 1000 rpm for 10 min at room temperature and then fixed in 2.5% glutaraldehyde for 1h. Then the cells were fixed in 1% osmium tetroxide solution for 1h, dehydrated in a series of ethanol washes (30, 60, 70, 90 and 100%), with that infiltrated the cells and embedded in LR White. Ultra-thin sections (50 - 75 nm) sliced with an LKB ultramicrotome were stained with 2% aqueous uranyl acetate and 2% aqueous lead citrate, and then photographed under 120 kV FEI Tecnai G2 Spirit TEM (FEI, Hongkong).

### Flow cytometry assay

In cell cycle analysis, cells in every group were digested with 0.25% trypsin. After 24 h treatment SiHa cells (4 μM DDP and 8 μM UA) were washed twice using cold PBS. Then, cells were re-suspended in prepared sample buffer containing 20 μL 5 μg/mL propidium iodide and 50 μL 10 mg/mL RNase A. Cells were then incubated in the dark for 10 min at 37 °C.

Annexin V-fluorescein isothiocyanate (Annexin V-FITC) and propidium iodide (PI, Sigma) apoptosis detection kit (Becton Dickinson, NJ, USA) were used to evaluate the apoptosis status of SiHa cells. Cells were re-suspended in binding buffer and mediated to a concentration level of 0.5∼1 × 10^6^/ml. The suspension (100 μl) was incubated with 5 μl of Annexin V-FITC and PI for 15 minutes at room temperature in the dark. After 400μl binding buffer was added into each tube, apoptotic cells were evaluated with flow cytometry (Beckman FC 500 MCL/MPL, USA).

### Western blot assay

The expression level of NF-κB p65, Bcl-2, Bax, Caspase-3, PARP, PARP cleavage, p21, cyclin D1 and cyclin E were examined by western blot. After 48-hour treatment, cellular proteins were extracted and nuclear extracts were obtained using the nuclear protein extraction kit (Beyotime Institute of Biotechnology, China). BCA method was adopted for protein concentration detection. An equal amount of proteins in each group was loaded and separated by sodium dodecyl sulfate-polyacrylamide gelelectrophoresis (SDS-PAGE). The proteins were then transferred onto polyvinylidene fluoride membranes (PVDF) and blocked with 5% non-fat milk. The membranes were then probed with primary antibodies against p65, Bcl-2, Bax, Caspase-3, PARP, PARP cleavage, p21, cyclin D1, cyclin E and glyceraldehyde-3-phosphate dehydrogenase (GAPDH) (mouse anti-human, CST, American) respectively at 4°C overnight,. Membranes were washed three times using TBST (10 min each). Horseradish-peroxidase-linked secondary antibodies (rabbit anti-mouse) were then added and membranes were incubated at room temperature for 1 h. After this, membranes were washed three times (10 min each) with TBST, and signal detection was carried out using Super ECL Plus Detection Reagent Kit (Applygen Technologies Inc., China).

### Statistical analysis

All statistical analyses were performed using SPSS 19.0 software. Significant differences in numerical data (mean ± SD) among different groups were compared using the Analysis of Variance (ANOVA). *P-*value < 0.05 indicated a statistical significance.

## SUPPLEMENTARY MATERIALS TABLES



## Abbreviations

(UA): Ursolic acid
(DDP): Cisplatin
(NF-κB): Nuclear factor-kappa B
(TEM): Transmission electron microscopy
(DMSO): Dimethyl sulfoxide
(STR): Short tandem repeat
(DMEM): Dulbecco’s Modified Eagles Medium
(cDNA): Complementary DNA
(RPMI): Roswell Park Memorial Institute
(PBS): Phosphate-buffered saline
(TEM): Transmission electron microscopy
(SDS-PAGE): Sulfate-polyacrylamide gelelectrophoresis
(PVDF): Polyvinylidene fluoride membranes
(GAPDH): Glyceraldehyde-3-phosphate dehydrogenase
(ANOVA): Analysis of variance
