# Supplementary material for: Synergism of ursolic acid and cisplatin promotes apoptosis and enhances growth inhibition of cervical cancer cells via suppressing NF-κB p65
Source: Oncotarget. 2017 Oct 30;8(57):97416–27. doi: 10.18632/oncotarget.22133 (PMC5722573; doi:10.18632/oncotarget.22133)
Supplement: Supplementary file 1 [file oncotarget-08-97416-s001.pdf]

Synergism of ursolic acid and cisplatin promotes apoptosis and enhances growth inhibition of cervical cancer cells via suppressing NF-κB p65

SUPPLEMENTARY MATERIALS

Supplementary Table 1: The percentage of apoptotic cells

Group	1	2	3	4	5	6	Mean ± SD
Control	13.78	13.45	13.66	13.37	13.20	12.78	13.37±0.36
DDP	37.80	37.69	39.63	38.52	36.74	39.52	38.32±1.13**
UA	24.85	31.04	29.84	29.35	31.78	32.08	29.82±2.66$^{**##}$
DDP+UA	60.32	62.13	55.38	59.43	59.44	57.85	59.09±2.29$^{**##\circ\circ}$

Results (mean ± SD) were from six independent experiments. ** P < 0.01 versus control group; $^{##}$ P < 0.01 compared with the DDP group; $^{\circ\circ}$ P < 0.01 compared with the UA group. DDP: cisplatin; UA: ursolic acid.

Supplementary Table 2: The quantifications of the protein expression levels

Group	Protein	1	2	3	4	5	6	Mean ± SD
Control	Caspase-3	0.79	0.89	1.09	0.75	1.09	1.40	1.00±0.24
	Bcl-2	1.46	1.09	0.85	0.59	0.88	1.12	1.00±0.30
	Bax	1.04	1.22	1.08	0.82	1.00	0.87	1.00±0.15
	PARP	1.00	1.14	0.92	1.29	0.78	0.91	1.00±0.18
	PARP cleavage	1.16	1.04	0.81	0.95	1.02	1.01	1.00±0.12
DDP	Caspase-3	1.71	2.03	2.57	1.98	2.07	2.14	2.08±0.28**
	Bcl-2	0.48	0.48	0.44	0.57	0.54	0.45	0.49±0.05*
	Bax	1.65	1.82	2.51	2.14	1.90	1.56	1.93±0.35**
	PARP	0.81	1.00	0.95	1.04	0.92	0.74	0.91±0.11
	PARP cleavage	2.09	1.91	1.74	2.01	1.84	1.55	1.86±0.20**
UA	Caspase-3	1.67	1.24	1.61	1.49	1.51	1.44	1.49±0.15$^{**##}$
	Bcl-2	0.48	1.01	0.87	0.47	0.78	0.69	0.72±0.21$^{*#}$
	Bax	1.56	1.40	1.64	1.30	1.49	1.63	1.50±0.13$^{**##}$
	PARP	1.16	0.83	0.99	0.82	1.01	0.91	0.95±0.13
	PARP cleavage	1.46	1.24	1.61	1.55	1.44	1.54	1.47±0.13$^{**##}$
DDP+UA	Caspase-3	2.82	2.92	3.25	2.18	3.01	3.46	2.94±0.44$^{**##\circ\circ}$
	Bcl-2	0.19	0.31	0.25	0.25	0.24	0.32	0.26±0.05$^{**##\circ\circ}$
	Bax	2.69	2.37	2.59	3.15	2.55	2.57	2.66±0.27$^{**##\circ\circ}$
	PARP	0.80	0.92	1.06	0.68	0.90	0.96	0.89±0.13
	PARP cleavage	2.34	2.72	1.99	2.27	2.07	2.19	2.26±0.26$^{**##\circ\circ}$

Results (mean ± SD) were from six independent experiments. * P < 0.05, ** P < 0.01 versus the control group; $^{#}$ P < 0.05, $^{##}$ P < 0.01 compared with the DDP group; $^{\circ}$ P < 0.05, $^{\circ\circ}$ P < 0.01 compared with the UA group. DDP: cisplatin; UA: ursolic acid.

Supplementary Table 3: The quantifications of expression levels of p21, cyclin E and D1

Group	Protein	1	2	3	4	5	6	Mean ± SD
Control	P21	1.03	1.13	0.84	1.04	0.81	1.15	1.00±0.14
	Cyclin E	0.90	1.18	0.94	1.00	0.91	1.08	1.00±0.11
	Cyclin D1	0.84	0.96	1.19	1.04	0.97	0.99	1.00±0.11
DDP	P21	2.41	2.56	2.82	2.24	2.08	2.14	2.42±0.29**
	Cyclin E	0.47	0.51	0.48	0.44	0.36	0.43	0.45±0.05**
	Cyclin D1	0.58	0.62	0.55	0.52	0.43	0.55	0.54±0.07*
UA	P21	1.92	2.43	1.93	2.06	1.89	1.92	2.05±0.22**##
	Cyclin E	0.71	0.67	0.63	0.72	0.81	0.78	0.71±0.07**#
	Cyclin D1	0.56	0.72	0.78	0.74	0.79	0.75	0.72±0.09*#
DDP+UA	P21	3.20	2.70	3.57	2.48	2.86	3.09	2.96±0.43**#°°
	Cyclin E	0.21	0.21	0.19	0.20	0.24	0.26	0.21±0.02**#°°
	Cyclin D1	0.29	0.27	0.33	0.27	0.31	0.26	0.30±0.03**#°

Results (mean ± SD) were from six independent experiments. * $P < 0.05$, ** $P < 0.01$ versus the control group; # $P < 0.05$, ## $P < 0.01$ compared with the DDP group; ° $P < 0.05$, °° $P < 0.01$ compared with the UA group. DDP: cisplatin; UA: ursolic acid.

Supplementary Table 4: The quantifications of the nuclear p65 expressions

Group	1	2	3	4	5	6	Mean ± SD
Control	1.02	0.90	0.96	1.25	0.95	0.94	1.00±0.13
DDP	1.12	1.25	1.46	1.14	1.44	1.16	1.26±0.15**
UA	0.63	0.46	0.43	0.60	0.44	0.69	0.54±0.11**##
DDP+UA	0.74	0.90	0.93	0.78	0.69	0.65	0.78±0.11*##°

Results (mean ± SD) were from six independent experiments. * $P < 0.05$, ** $P < 0.01$ versus the control group; # $P < 0.05$, ## $P < 0.01$ compared with the DDP group; ° $P < 0.05$, °° $P < 0.01$ compared with the UA group. DDP: cisplatin; UA: ursolic acid.
